# Whole-Genome Sequencing: The Key to Unlocking a Nosocomial Outbreak of Coronavirus Disease 2019 (COVID-19)

**DOI:** 10.1017/ash.2021.98

**Published:** 2021-07-29

**Authors:** Lindsey Gottlieb, Emilia Sordillo, Harm van Bakel, Barbara Smith, Bernard Camins, Sarah Alsamarai, Viviana Simon, Kilyoub Kim

## Abstract

**Background:** Accurately tracing nosocomial transmission of coronavirus disease 2019 (COVID-19) is critical to developing effective infection prevention policies. Given the high prevalence and variable incubation period of SARS-CoV-2 infection, the utility of traditional contact tracing is limited. We describe a nosocomial outbreak in which whole-genome sequencing (WGS) was pivotal to identifying the primary case. **Methods:** This study was conducted at a New York City academic hospital. The index case was identified on August 13, 2020, and the last case on September 9, 2020. Hospital policy required all inpatients to be screened for COVID-19 on admission by SARS-CoV-2 molecular amplification testing. All healthcare workers (HCWs) were required to wear masks and eye protection for patient care. After a patient (patient 1), who tested SARS-CoV-2 negative on admission, was positive on preprocedure screening on hospital day 9, contact tracing was initiated. Two patients (patients 2 and 3) and 13 HCWs with high-risk exposures (HREs) to patient 1 were quarantined and referred for testing. Additional surveillance testing was performed on 18 inpatients and 84 HCWs on the affected unit. Patients 2 and 3 and 3 HCWs (HCW-1, -2, and -3), only 1 of whom had a high-risk exposure to patient 1, tested positive. WGS was performed to further investigate this outbreak. **Results:** The outbreak variant (clade 20A) was found in samples from 6 patients and 2 HCWs. Patients 2 and 3 were roommates of patient 1 in the 2 days before patient 1’s positive test, and they did not consistently wear masks in the room. HCW-1 placed a peripheral IV in patient 1 the day before patient 1’s positive test without wearing eye protection. Four additional cases in this cluster (patients 4–6 and HCW-4) were identified by surveillance WGS of positive tests. A review indicated that patient 1 was located ~3 m (~10 feet) away from patient 4 in the emergency department (ED) for 6 hours on hospital day 1, when the admission SARS-CoV-2 test from patient 4 was not positive. No epidemiologic link was found to patient 5 or 6 or HCW-4. The specimen from HCW-2 was inadequate for WGS. The specimen from HCW-3 was not linked to this cluster. **Conclusions:** This complex nosocomial outbreak highlights the importance of WGS in understanding transmission events. Patient 4 was not identified by traditional contact tracing but was linked to patient 1 and was recognized as the primary case through WGS, having likely infected patient 1 in the ED. Based on these findings, we focused our corrective actions on more promptly isolating suspected COVID-19 cases in the ED, increasing inpatient masking, and improving HCW adherence to universal eye protection.

**Funding:** No

**Disclosures:** None

